# Exploration of genetically encoded voltage indicators based on a chimeric voltage sensing domain

**DOI:** 10.3389/fnmol.2014.00078

**Published:** 2014-09-29

**Authors:** Yukiko Mishina, Hiroki Mutoh, Chenchen Song, Thomas Knöpfel

**Affiliations:** ^1^Laboratory for Neuronal Circuit Dynamics, RIKEN Brain Science InstituteWako, Japan; ^2^Centre for Global Communication Strategies, The University of TokyoTokyo, Japan; ^3^Department of Neurophysiology, Hamamatsu University School of MedicineShizuoka, Japan; ^4^Division of Brain Sciences, Department of Medicine, Imperial College LondonLondon, UK

**Keywords:** optogenetics, mouse model, optical imaging, voltage imaging, FRET sensors

## Abstract

Deciphering how the brain generates cognitive function from patterns of electrical signals is one of the ultimate challenges in neuroscience. To this end, it would be highly desirable to monitor the activities of very large numbers of neurons while an animal engages in complex behaviors. Optical imaging of electrical activity using genetically encoded voltage indicators (GEVIs) has the potential to meet this challenge. Currently prevalent GEVIs are based on the voltage-sensitive fluorescent protein (VSFP) prototypical design or on the voltage-dependent state transitions of microbial opsins. We recently introduced a new VSFP design in which the voltage-sensing domain (VSD) is sandwiched between a fluorescence resonance energy transfer pair of fluorescent proteins (termed VSFP-Butterflies) and also demonstrated a series of chimeric VSD in which portions of the VSD of *Ciona intestinalis* voltage-sensitive phosphatase are substituted by homologous portions of a voltage-gated potassium channel subunit. These chimeric VSD had faster sensing kinetics than that of the native Ci-VSD. Here, we describe a new set of VSFPs that combine chimeric VSD with the Butterfly structure. We show that these chimeric VSFP-Butterflies can report membrane voltage oscillations of up to 200 Hz in cultured cells and report sensory evoked cortical population responses in living mice. This class of GEVIs may be suitable for imaging of brain rhythms in behaving mammalians.

## INTRODUCTION

Deciphering how the brain generates cognitive function from patterns of electrical signals is one of the ultimate challenges in neuroscience. Advances toward this goal require a better understanding of the “neuronal code,” and being able to monitor electrical signals of very large neuronal populations with fine temporal resolution is central to the progress. Even when restricted to relatively simple behaviors (such as goal-directed motor actions in response to sensory stimuli), observing only cortical activities, and in small animals such as mice, the monitoring of electrical activities must cover mesoscopic areas of tissue (dimensions measured at the millimeter scale). Optical voltage imaging methods have, at least in principle, the spatio-temporal resolution necessary for this endeavor (Ross et al., 1974; Grinvald et al., 1977; Grinvald and Hildesheim, 2004). In particular, voltage-sensitive dyes have been fruitfully employed in widefield epifluorescence imaging (Shoham et al., 1999; Petersen et al., 2003a; Grinvald and Hildesheim, 2004), and this approach has contributed much to the understanding of cortical circuit dynamics, especially in visual and somatosensory areas (Shoham et al., 1999; Kenet et al., 2003; Petersen et al., 2003a,b; Grinvald and Hildesheim, 2004).

Recently developed genetically-encoded voltage indicators (GEVIs) promise to improve upon classical voltage-sensitive dyes in at least four aspects: (i) they allow for non-invasive transcranial imaging in species with thin craniums (such as mice), which eliminates the previously compulsory craniotomies for dye staining; (ii) they provide reliable recordings from the same neuronal population in a subject over prolonged periods of time for multiple sessions; (iii) they genetically target specific cell populations, so the signals originate only from specific neurons of interest in an otherwise diverse population; (iv) they enable transgenic expression strategies that provide highly reproducible expression of protein indicators in different animals to eliminate between-subject variability.

There are two classes of conceptual designs of GEVIs currently being pursued. The first type is the microbial opsin-based GEVIs that exhibit voltage-dependent state-transitions in their photocycles (Kralj et al., 2012; Maclaurin et al., 2013). These opsin-based probes were initially limited by their low brightness (Mutoh et al., 2012; Mutoh and Knöpfel, 2013), but this issue has been successfully addressed in very recent work (Gong et al., 2014; Zou et al., 2014). The second type is the voltage-sensitive fluorescent protein (VSFP) class of GEVIs. These utilize the voltage-dependent structural rearrangement of voltage-sensing domains (VSDs), which are homologous to the S1–S4 transmembrane segment of Kv potassium channels. Thus far, several VSFP derivatives have enabled voltage imaging in brain slices as well as in intact mouse brain (Akemann et al., 2010, 2012; Mutoh et al., 2012; St-Pierre et al., 2014).

The first VSFPs (VSFP1 and VSFP2.x) exploited the voltage-dependent VSD structural rearrangement to modulate fluorescence resonance energy transfer (FRET) efficacy between a tandem pair of fluorescent proteins (Sakai et al., 2001; Dimitrov et al., 2007). Dissection of a FRET independent component of the voltage response led to the development of the monochromatic (single fluorescent protein) VSFP3.x (Lundby et al., 2008; Perron et al., 2009a,b). More recently, we introduced VSFP-Butterflies, in which two fluorescent proteins are positioned so that the VSD is now sandwiched between the FRET pair (Akemann et al., 2012). These VSFP-Butterflies permitted imaging of sub-threshold activity *in vivo* in specific neuronal populations in awake behaving mice (Akemann et al., 2012). The VSFP2.x, VSFP3.x, and the VSFP-Butterfly scaffolds were adopted for other fluorescent proteins (Tsutsui et al., 2008, 2013; Jin et al., 2012).

The first VSFP with robust signals in mammalian cells used the voltage sensor of *Ciona intestinalis* voltage-sensitive phosphatase (Ci-VSP) whose VSD is homologous to that of Kv potassium channels (VSFP2.1; Dimitrov et al., 2007). Subsequent VSFP type of GEVIs [e.g., VSFP2.3 and VSFP3.1 (Lundby et al., 2008); VSFP2.4 (Akemann et al., 2010); VSFP-mUKG-mKOκ (Tsutsui et al., 2008); VSFP-CR (Lam et al., 2012); ArcLight (Jin et al., 2012)], and ASAP1 (St-Pierre et al., 2014) generally substituted different fluorescent proteins or VSDs and varied the linking arrangements of the two components.

In order to overcome the limited response kinetics of current VSFPs, we developed chimeric VSDs in which portions of the Ci-VSP VSD was replaced by homologous portions of the Kv3.1 voltage-gated potassium channel subunit (Mishina et al., 2012). Insertion of these chimeric VSDs into the VSFP2.3 scaffold led to a series of chimeric VSFP variants, many of which efficiently target to the membrane of PC12 and human embryonic kidney (HEK) cells and exhibit optimized kinetics which retained Kv3.1 characteristics.

Here, we describe a new set of VSFPs that combine the chimeric VSDs with the VSFP-Butterfly structure. We show that these chimeric VSFP-Butterflies can report membrane voltage oscillations of up to 200 Hz in cultured cells and report sensory evoked cortical population responses in living mice. These variants of GEVIs may be suitable for imaging of brain rhythms in awake, behaving mammals.

## MATERIALS AND METHODS

### MOLECULAR BIOLOGY

**FIGURE 1:**
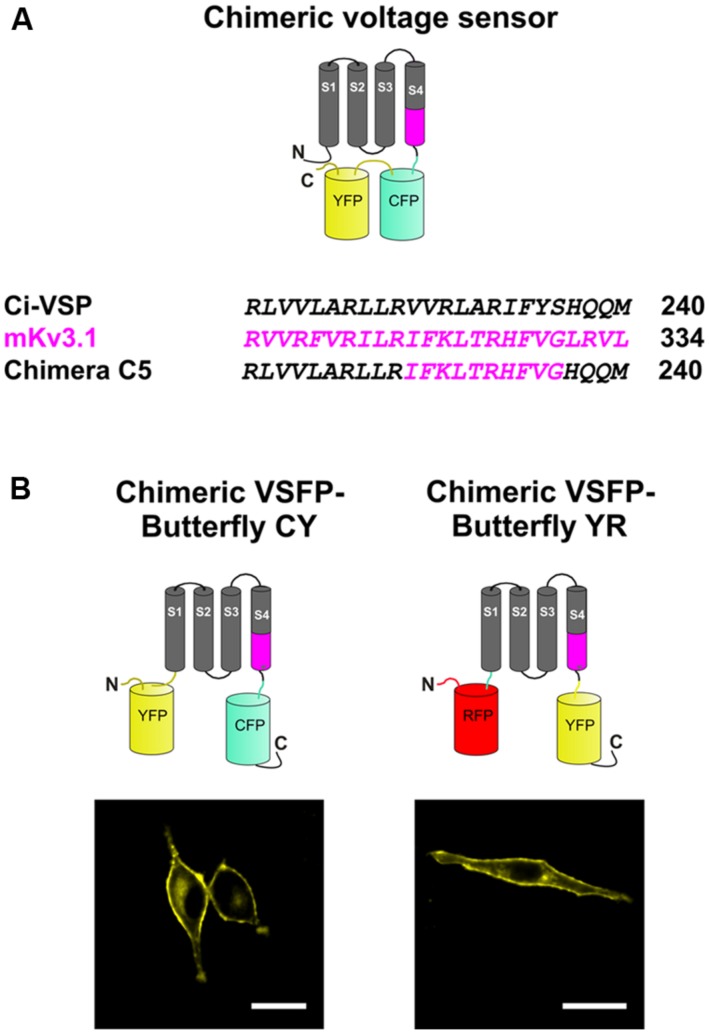
**Design of a chimeric voltage sensor by transferring a fragment of Kv3.1 to *Ciona intestinalis* voltage-sensitive phosphatase (Ci-VSP) in the S4 transmembrane segment. (A)** Schematic depiction **(top)** of a chimeric voltage sensor, Chimera C5, in which a section of the voltage-sensing domain (VSD) of Ci-VSP is exchanged with amino acids from the S4 transmembrane segment of Kv3.1 **(bottom)**. The fluorescent proteins that are attached to the C-terminus of the chimeric construct enables visualization of membrane targeting and monitoring of voltage-dependent fluorescence changes due to movements of the VSD. **(B)** Modifications in the pair of the fluorescent proteins and localization of the constructs at the plasma membrane. The donor fluorescent protein was kept at the C-terminus, whereas the acceptor fluorescence was attached to the N-terminus. The donor/acceptor pairs were mCerulean/mCitrine for Chimeric voltage-sensitive fluorescent protein (VSFP)-Butterfly cyan–yellow (CY; **left)** and mCitrine/mKate2 for Chimeric VSFP-Butterfly yellow–red (YR; **right)**. Scale bar, 20 μm.

The chimeric Butterfly constructs were based on previously published versions of VSFPs, namely a combination of Chimera C5 (Mishina et al., 2012), in which a region of the VSD of Ci-VSP was substituted with that of the Kv3.1 potassium channel and VSFP-Butterfly 1.2 (Akemann et al., 2012; **Figure 1**). Both Chimeric VSFP-Butterfly cyan–yellow (CY; mCerulean/mCitrine) and Chimeric VSFP-Butterfly yellow–red (YR; mCitrine/mKate2) were generated using sequential polymerase chain reactions following the previously published protocols (Lundby et al., 2008; Mutoh et al., 2009; Akemann et al., 2012; Mishina et al., 2012). Briefly, Chimeric VSFP-Butterfly YR was generated by substituting the Ci-VSP VSD sequence of VSFP-Butterfly 1.2 (Akemann et al., 2012) with that of Kv3.1 VSD. This was performed by introducing restriction sites (XhoI and EcoRV) at the terminal ends of the VSD in both VSFP-Butterfly 1.2 and Chimera C5 (Mishina et al., 2012) as silent mutations and substituting the Chimera C5 VSD into the VSFP-Butterfly 1.2. In addition, a single mutation, K234R of mKate2, was introduced by site-directed mutagenesis for decreased intracellular aggregation and enhanced brightness (Perron and Knöpfel, unpublished observations). Chimeric VSFP-Butterfly CY was designed to incorporate the mCerulean/mCitrine fluorescence reporters, rather than the mCitrine/mKate pair (Mutoh et al., 2009). Similar to the VSFP-Butterfly 1.0 (Akemann et al., 2012), the mCitrine FRET acceptor was attached to the VSD at position 70 by overlap extension polymerase chain reactions after removal of the mCitrine of VSFP2.3. All constructs were subsequently subcloned into both pcDNA3.1(-; for functional imaging in cell culture) and pCAG vectors (for *in vivo* imaging; Lundby et al., 2008; Akemann et al., 2012) by utilizing NheI and AflII restriction endonucleases. DNA sequences for all of the constructs were confirmed by DNA sequencing analysis.

Chimeric VSFP-Butterfly CY and chimeric VSFP-Butterfly YR are deposited at Addgene (pCAG-Chimeric_Butterfly_CY_1.0, 59800; pCAG-Chimeric_Butterfly_YR_1.0, 59801).

### CELL CULTURE, *IN VITRO* OPTICAL IMAGING, AND *IN UTERO* ELECTROPORATION

PC12 cells were cultured in Dulbecco’s modified Eagle’s medium supplemented with 10% horse serum, 5% fetal bovine serum, and 1% penicillin and streptomycin (GIBCO) at 37°C. HEK293T cells were cultured in Dulbecco’s modified Eagle’s medium supplemented with 10% fetal bovine serum and 1% penicillin and streptomycin (GIBCO) at 37°C. Cells were grown on poly-D-lysine coated coverslips and transfected 24 h after plating using Lipofectamine 2000 reagent (Invitrogen) and washed daily. Experiments were performed 2–3 days after transfection. PC12 cell images were obtained with a confocal laser scanning microscope (C1si/FN1, Nikon) for expression screening. *In utero* electroporation were performed as previously described (Akemann et al., 2010).

### ELECTROPHYSIOLOGY AND FUNCTIONAL OPTICAL IMAGING

The voltage clamp recordings were performed on the instrumental set-up as previously described (Akemann et al., 2010, 2012). Briefly, voltage-dependent fluorescence recordings from both PC12 and HEK cells were performed by combining voltage clamp (under the whole-cell configuration of the patch-clamp technique) with dual-emission microfluorometry. Electrical and optical data were acquired using pCLAMP 10.1 software (Axon Instruments). PC12 or HEK cells were continuously perfused (1.5–2 ml/min) with a bathing solution containing (in mM) 150 NaCl, 4 KCl, 2 CaCl_2_, 1 MgCl_2_, 5 Glucose, 5 HEPES (pH 7.4 with NaOH). Patch electrodes had resistances of 3–5 MΩ when filled with intracellular solution containing (in mM) 130 CsCl, 1 MgCl_2_, 20 HEPES, 5 EGTA, 3 MgATP (pH 7.2 with CsOH). All data were low-pass filtered with a cutoff frequency of 5 kHz and digitized at 5 kHz using a Digidata 1322 analog-to-digital converter (Axon Instruments). Fluorescence was illuminated by light from a computer-controlled monochromator (Polychrome IV, T.I.L.L. Photonics). For VSFP2.3, Chimera C5 and Chimeric VSFP-Butterfly CY, excitation light (440 nm) was reflected and first passed through a 458-nm dichroic mirror (FF458-Di01, Semrock). Emitted fluorescence was then split by a 506-nm dichroic mirror (FF506-Di03, Semrock) onto two photodiodes (T.I.L.L. Photonics) behind Cerulean- and Citrine- specific filters (BP 482 ± 35 nm: FF01-482/35-25 and LP 514 nm: LP02-514RU-25, Semrock). For Chimeric VSFP-Butterfly YR, excitation light (488 nm) was reflected and first passed through a 506-nm dichroic mirror (FF506-Di03, Semrock). Emitted fluorescence was then split by a 593-nm dichroic mirror (FF593-Di03, Semrock) onto two photodiodes (T.I.L.L. Photonics) behind Citrine- and mKate2- specific filters (BP 542 ± 13.5 nm: FF01-542/27-25 and LP 594 nm: BLP01-594R-25, Semrock).

**FIGURE 2:**
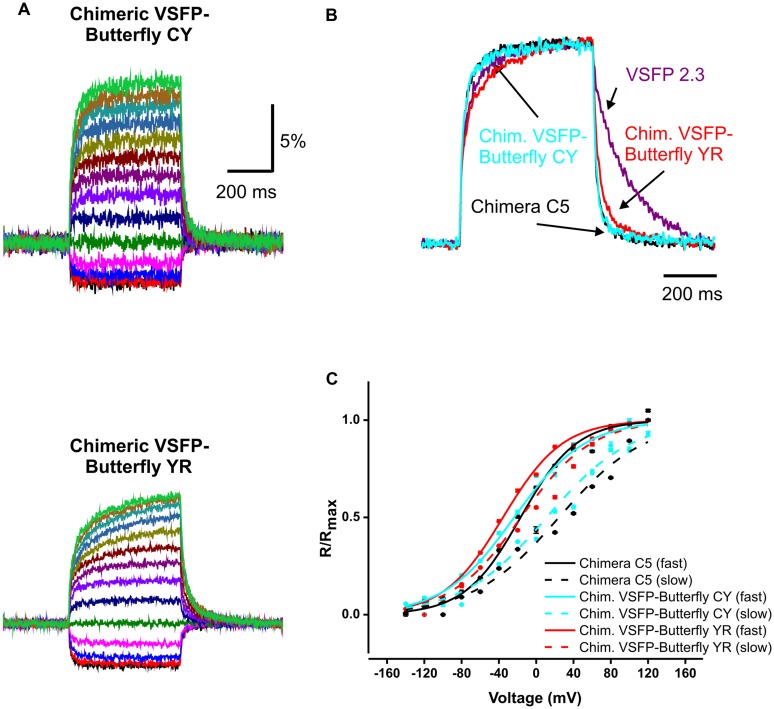
**Kinetics of voltage dependency of Chimeric VSFP-Butterfly CY and Chimeric VSFP-Butterfly YR in PC12 cells. (A)** Comparison of ratiometric fluorescence signals of Chimeric VSFP-Butterfly CY **(upper)** and Chimeric VSFP-Butterfly YR **(lower)** in response to a family of voltage steps from a holding potential of -60 mV, and 20 mV step increments from -140 to 120 mV. (*n* = 6). **(B)** Normalized responses to depolarization from -60 to 20 mV and responses to return for VSFP2.3 (magenta), Chimera C5 (black), Chimeric VSFP-Butterfly CY (blue), and Chimeric VSFP-Butterfly YR (red). **(C)** Amplitude of normalized optical signal (ΔR/R) versus membrane voltage for the fast (solid line) and slow (dotted line) components of Chimera C5 (black), Chimeric VSFP-Butterfly CY (blue), and Chimeric VSFP-Butterfly YR (red). Responses (ΔR/R) to families of voltage steps (as in **A**) were fitted with the function *y* = y0 + A1*exp (-t/τ1_on) + A2*exp (-t/τ2_on) where t is time, τ1_on the faster on time constant and τ2_on the slower on time constant. A1 and A2 are the amplitudes of fast and slow response components and are plotted against the step voltage. *N* = 6–7; error bars ± SEM.

The following protocol was used to test the voltage-dependence of the constructs (**Figure 2**). From a holding potential of -60 mV, cells were held for 500 ms at voltages between -140 mV and 120 mV in 20 mV steps to elicit fluorescence signals from mCerulean and mCitrine (VSFP2.3, Chimera C5, and Chimeric VSFP-Butterfly CY) or mCitrine and mKate2 (Chimeric VSFP-Butterfly YR). Finally, to test the frequency response of the constructs, sinusoidal voltage oscillations (20 mV amplitude) from -70 were generated in voltage clamp mode at frequencies of 10, 50, 100, and 200 Hz.

For all optical data, background data were obtained from a region on the coverslips devoid of fluorescent proteins. Photobleaching was corrected by division of a double exponential fit of the fluorescence trace at the holding potential. The ratiometric fluorescent signals were obtained by dividing the signals from the two fluorescent proteins. Calculations, including time constants, dynamic range, and V_1/2_ were calculated as previously described (Akemann et al., 2012). Data are expressed as mean ± SEM, with *n* specifying the number of independent experiments. For each cell and voltage protocol 6–10 traces were averaged.

*In vivo* optical imaging was performed on mice (over 60 days old) *in utero* electroporated with expression plasmids. A glass window was implanted under surgical anesthesia (pentobarbital by intraperitoneal injection) as previously described (Akemann et al., 2012). Briefly, after exposing the cranial bone by removing the soft tissue, the left somatosensory cortex was carefully thinned using a dental drill and a cover glass was mounted over the cortex. A metal bolt was fixed on the frontal–medial cranium with dental cement. For imaging, mice were mounted onto a custom-made stereotaxic frame for stabilization and the body temperature was kept at 37°C (Fine Science Tools, Tokyo). The stimulus to the contralateral whisker C1 was delivered by a focal air puff system (100 ms, Picospritzer III, Parker Hannifin). Dual-emission optical imaging was performed using two synchronized CCD cameras (Sensicam, PCO) at 50 frames/s. Excitation light was provided by a high-power halogen lamp (Moritex). The following filters and splitters were used for optical recording: mCitrine excitation (FF01 483/32-25), mCitrine emission (F01 542/27-25), mKate2 emission (BLP01 594R-25), LP506 nm (FF 506-Di03) as excitation beam splitter, and LP593 nm (FF 593-Di03) as detection splitter, all installed onto the THT macroscopy system (Brainvision, Tokyo). Images were acquired using a custom-made macros using ImagePro6.2.

Animal experiments were performed under the Institutional Animal Care and Use Committee of the RIKEN Wako Research Center.

## RESULTS

### CHIMERIC VSFP-BUTTERFLIES

We previously systematically replaced portions of the Ci-VSP VSD with homologous sections of the Kv3.1 potassium channel subunit, yielding chimeric VSDs that we termed Chimera Cx (x running from 1 to 40) and demonstrated that this replacement leads to an acceleration of voltage sensing movements in a subgroup of constructs (Mishina et al., 2012). For the present study, we used the construct Chimera C5, in which 10 consecutive amino acid segments from the C-terminal portion of the S4 of Kv3.1 replaced the homologous region in VSFP2.3 (**Figure 1A**).

The choice of the fluorescent protein pair for FRET measurements and the positioning relative to the VSD can significantly affect the biophysical properties of FRET-based genetically encoded indicator proteins (Alford et al., 2013). By moving the FRET acceptor from the C-terminus of the donor to the N-terminal end of the VSD, we generated and characterized two new Butterfly variants of the Chimera C5 construct. The two Butterfly variants were designed with mCerulean/mCitrine (“CY”) and mCitrine/mKate2 (“YR”) fluorescent protein FRET pairs (**Figure 1B**) and they were named Chimeric VSFP-Butterfly CY and Chimeric VSFP-Butterfly YR, respectively.

We first tested whether the VSFP-Butterfly conformation preserved the kinetic properties found for Chimera C5 described in Mishina et al. (2012; **Figure 2**; **Table 1**). We found no significant (*t*-test, *p* = 0.1–0.5; **Table 1**) difference between chimera C5 and Chimeric VSFP-Butterfly CY in terms of the response time constants and the overall ratiometric signal amplitudes. The Chimeric VSFP-Butterfly YR version exhibited a slightly larger second on time constant and a slower off time constant when compared to Chimera C5 (*t*-test, *p* < 0.001 for both, **Table 1**).

**Table 1:** Summary of the FRET response properties of chimeric voltage-sensitive fluorescent protein (VSFP)-Butterflies and previously published VSFPs.

	τ_1_ on	τ_2_ on	% τ_1_	τ off	V_1/2_ fast (mV)	V_1/2_ slow (mV)	ΔR/R (%)^#^
Chimeric VSFP-Butterfly cyan–yellow (CY)	2.1 ± 0.2	36.7 ± 1.1	60.0	14.6 ± 0.5	-24.0 ± 0.5	≥20	14.7 ± 0.2
Chimeric VSFP-Butterfly yellow–red (YR)	2.3 ± 0.2	81.2 ± 2.7	55.4	25.1 ± 0.9	-33.7 ± 0.3	-9.1 ± 0.4	12.7 ± 0.1
Chimera C5*	2.1 ± 0.2	36.8 ± 0.9	60.1	13.4 ± 0.5	-17.9 ± 0.4	≥20	14.8 ± 0.1
VSFP2.3*	3.0 ± 0.2	69.2 ± 1.8	26.6	91.6 ± 1.7	-28.3 ± 0.5	-48.6 ± 0.5	15.2 ± 0.1
Butterfly 1.2**	1.0 ± 0.7	12.2 ± 0.7	40.0	89.9 ± 5.2	-79 ± 2	-58.2 ± 5.3	15.0 ± 0.7

*From Mishina et al. (2012); **From Akemann et al. (2012); ^#^% ΔR/R values were obtained from the maximal and minimal acceptor/donor florescence ratio responses between -140 and 120 mV from a holding potential of -70 mV.

*Time constants τ on was calculated from a response from -70 mV holding potential to a 60 mV step, and τ off was calculated from a return to -70 mV. *n* = 6–9; Errors ± SEM.*

One of the advantageous features of our previous VSFP Butterfly 1.2 was a left shifted V_1/2_ value (as compared to its precursor VSFP2.42), increasing the sensitivity around the resting membrane potential (Akemann et al., 2012). A significant left shift of the V_1/2_ values was found for Chimeric VSFP-Butterfly YR when compared to Chimera C5 (*t*-test, *p* < 0.001; **Table 1**). A clear improvement of the Chimeric VSFP-Butterfly YR as compared to VSFP Butterfly 1.2 was a significant shortening of the off time constant (25.1 ± 0.9 versus 89.9 ± 5.2 ms; Akemann et al., 2012, *p* < 0.005). The faster off response was even more pronounced for Chimeric VSFP-Butterfly CY (14.6 ± 0.5, significant smaller than the value of Chimeric VSFP-Butterfly YR, *t*-test, *p* < 0.001; **Table 1**).

These characteristic parameters are summarized in **Table 1** along with previous published measurements for VSFP2.3 (Lundby et al., 2008, 2010; Akemann et al., 2010) and VSFP Butterfly 1.2 (Akemann et al., 2012).

### RESPONSES OF THE CHIMERIC VSFP-BUTTERFLY CONSTRUCTS TO OSCILLATORY MEMBRANE VOLTAGES

**FIGURE 3:**
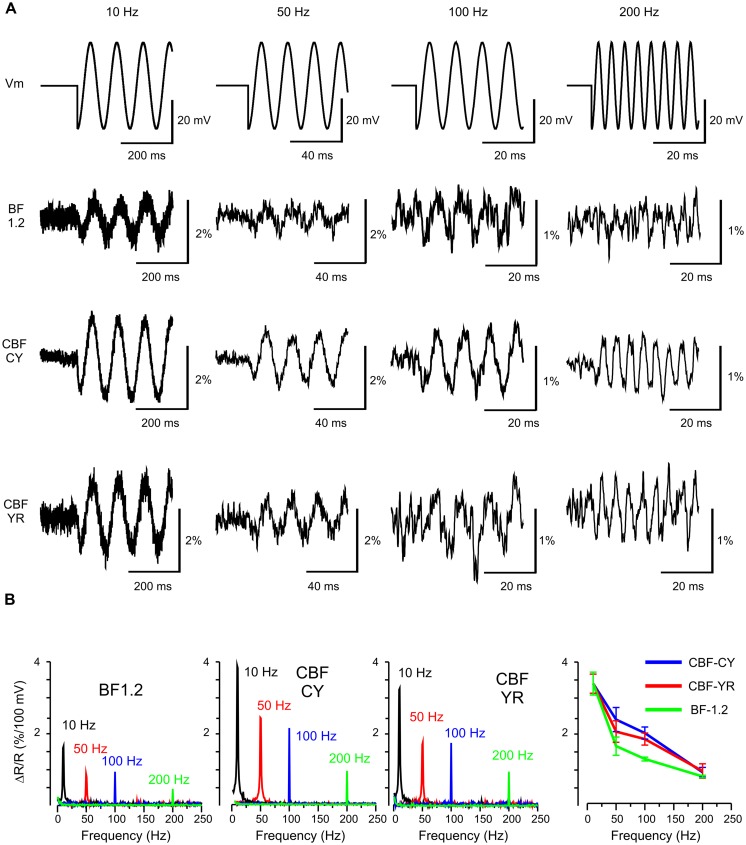
**Frequency response of VSFP Butterfly constructs to membrane voltage oscillations at 10, 50, 100, and 200 Hz in human embryonic kidney (HEK) cells. (A)** The upper row shows the command waveform of 20 mV oscillations around -70 mV. The second to fourth row depict the VSFP-Butterfly 1.2 (BF 1.2) Chimeric VSFP-Butterfly CY (CBF CY) and the Chimeric VSFP-Butterfly YR (CBF YR) ratiometric response signals (example recordings from one cell for each construct; average over 3–30 sweeps). **(B)** First three panels: Amplitude spectrograms for each oscillation frequency for each construct. Right panel: mean and SEM of the optical response at different frequencies.

To evaluate the use of chimeric VSFP-Butterflies for the potential to image brain rhythms in behaving mammalians, optical responses to oscillatory membrane potential were studied. To this end, PC12 and HEK cells were voltage clamped with an oscillatory voltage command. These experiments show that chimeric VSFP-Butterflies follow sinusoidal membrane voltage oscillations of up to 200 Hz, confirming the above initial assessment of these constructs demonstrating optimized kinetics relative to VSFP-Butterfly 1.2 (Akemann et al., 2012; **Figure 3A**). Compared to VSFP-Butterfly 1.2 (Akemann et al., 2012), chimeric VSFP-Butterflies show a larger gain (sensitivity) at frequencies between 10 and 200 Hz (*p* < 0.05, *t*-test; **Figure 3**). The less pronounced loss of gain observed with chimeric VSFP-Butterflies between steady state sensitivity and report of oscillatory membrane voltage fluctuations (at 10 Hz or higher frequencies) can be explained by the larger contribution of the faster component of the on response (τ1 on) and the much faster off time constant (τ1 off; **Table 1**).

### *IN VIVO* DEMONSTRATION OF THE FUNCTIONAL POTENTIALS OF THE CHIMERIC CONSTRUCTS

**FIGURE 4:**
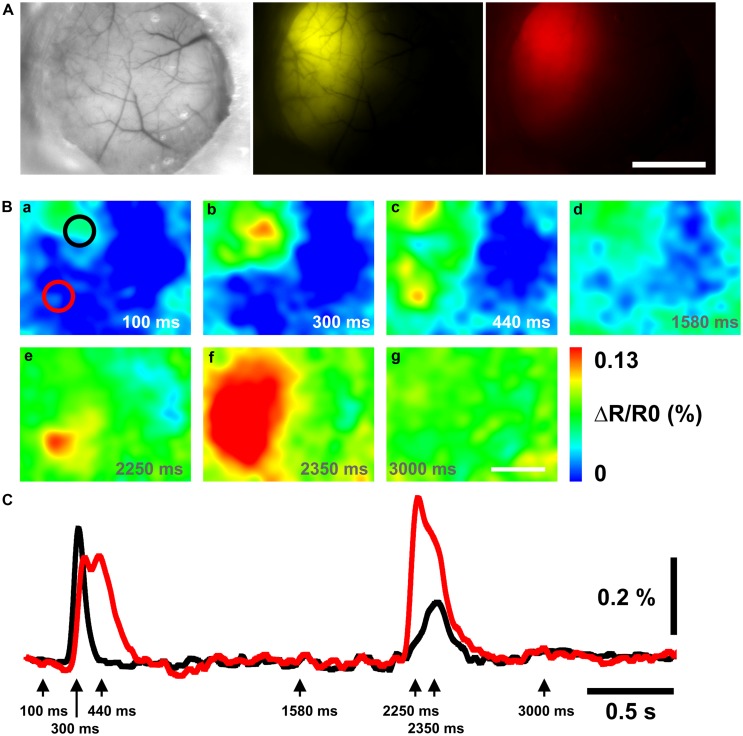
**Chimeric VSFP-Butterfly YR voltage imaging of cortical responses to whisker deflection and light flash. (A)** Brightfield **(left)** image and the expression pattern of mCitrine **(middle)** and mKate2 **(right)** of the left hemisphere. **(B)** Ratiometric images obtained before and after a single deflection of the C1 whisker and a brief flash of light. Scale bar, 2 mm. **(C)** Black and red traces of the ratiometric signal are taken from the black (somatosensory cortex) and red (visual cortex) circles in **(B-a)**, respectively. The arrows indicate the time points of the ratiometric images in **(B)**.

Chimeric VSFP-Butterfly YR was taken as a representative chimeric construct to explore performance *in vivo.* A relatively large transcranial window covering the left hemisphere of mice electroporated with the Chimeric VSFP-Butterfly YR construct exhibited strong fluorescence signals (**Figure 4A**). Under anesthesia, the signal of the Chimeric VSFP-Butterfly YR on the somatosensory cortex could be induced from baseline (**Figure 4B-a**) by a single C1 whisker deflection (**Figure 4B-b**) and the signal propagated to other discrete cortical areas (motor cortex and secondary somatosensory cortex, **Figure 4B-c**), and eventually faded (**Figure 4B-d**). Stimulation using brief flashes of light also activated the visual cortex area (**Figure 4B-e**) which propagated through the hemisphere toward the rostral area (**Figure 4B-f**) also then gradually faded (**Figure 4B-g**). **Figure 4C** shows how those voltage signals propagated in two distinct cortices, the somatosensory cortex and the visual cortex.

## DISCUSSION

We generated and characterized a new set of VSFPs that combine a chimeric VSD with the VSFP-Butterfly structure. We show that these chimeric VSFP-Butterflies can report membrane voltage oscillations of up to 200 Hz in cultured cells. We also demonstrate that these GEVIs report sensory evoked cortical population responses in living mice. The *in vivo* assay is particularly important as some previously reported VSFP derivatives (e.g., “Mermaid”) were functional in transfected cultured cells but failed to provide robust voltage signals in equivalent *in vivo* assays (Akemann et al., 2012). Similarly, it remains to be seen whether the recently reported monochromatic green fluorescent VSFPs with high *in vitro* sensitivity such as ASAP1 (St-Pierre et al., 2014) are suitable for rodent *in vivo* imaging experiments where modulation of green indicator fluorescence is significantly affected by hemodynamic effects (Diez-Garcia et al., 2007) and where movement artifacts are a major challenge.

The present study addresses the need for GEVIs with fast kinetics. Previous work has indicated that the kinetics of FRET-based VSFP is limited by properties of the voltage-sensitive protein that undergoes a slower state transition into a relaxed state after an initial, relatively fast on response (Lundby et al., 2010). To overcome this kinetic limit, VSDs from various species have also been explored. In particular, the chick homolog of Ci-VSP appears to provide a scaffold that lead to VSFPs with faster kinetics (Han et al., 2013; St-Pierre et al., 2014). The advantage of chimeras between Ci-VSP and Kv potassium channel subunits, as introduced and employed here, is that Kv channels are among the best studied membrane proteins with a wealth of structural understanding (Villalba-Galea et al., 2008; Vargas et al., 2012). This will likely instigate a more rational approach in the fine-tuning of the voltage sensing portion of GEVIs.

Improvements of GEVIs will continue as they are essential tools in studies where processing of synaptic inputs and action potentials at frequencies greater than 10 Hz are of interest. We expect that voltage imaging will be instrumental when linking neuronal information flows across large cortical areas and complex behavior GEVIs are likely on their way to replace the current low molecular weight voltage-sensitive dyes used in such long-term mesoscopic (circuit-centric) imaging approaches (Knöpfel, 2012). In this brain mapping research domain, GEVIs will become central to bridging the gap between single-neuron and whole-brain recordings as they are able to target specific cell populations and are able to provide consistent recordings over extended periods at the same locations.

The new VSFPs reported here outperform previously published GEVIs with demonstrated *in vivo* performance that are based on coupling a VSD with a FRET pair of fluorescent proteins by a factor of two in response time while keeping former advantages and strengths such as utilizing ratiometric outputs and brightness necessary for robust, high-resolution signals. Future fine-tuning of the chimeric Butterflies introduced here may include further enhancements in VSD kinetics and dynamic range, and may involve varied fluorescent proteins for increased brightness and photostability.

## Conflict of Interest Statement

The Associate Editor Katsuhiko Mikoshiba declares that, despite being affiliated to the same institution as authors, the review process was handled objectively and no conflict of interest exists. The authors declare that the research was conducted in the absence of any commercial or financial relationships that could be construed as a potential conflict of interest.
